# “Quite Devastating, Traumatic, and Lonely”: Understanding the Mental Health Experiences of Hispanic/Latino Rectal Cancer Patients and Their Caregivers

**DOI:** 10.1002/cam4.71253

**Published:** 2025-09-16

**Authors:** Zaria N. Cosby, Julian P. Howland, Eleanor Brown, Lucas K. Carpenter, Kristen M. Davis‐Lopez, Min Young Kim, Patricia Castañeda, Maria Gonzalez, Miriam T. Hernandez, Ysabel Duron, Gladys M. Rodriguez, Sandra S. Zaky, Arden M. Morris, Aaron J. Dawes

**Affiliations:** ^1^ Department of Surgery Stanford University School of Medicine Stanford California USA; ^2^ Stanford‐Surgery Policy Improvement Research and Education Center Stanford California USA; ^3^ Department of Surgery Icahn School of Medicine at Mount Sinai New York New York USA; ^4^ Department of Radiation Oncology Stanford University School of Medicine Palo Alto California USA; ^5^ Brigham Young University Provo Utah USA; ^6^ Oakland University William Beaumont School of Medicine Rochester Michigan USA; ^7^ Visión y Compromiso Los Angeles California USA; ^8^ The Latino Cancer Institute San Jose California USA; ^9^ Division of Hematology and Oncology Northwestern University Chicago Illinois USA

**Keywords:** caregivers, colorectal cancer, distress, Hispanic/Latino, mental health, qualitative, secondary analysis

## Abstract

**Background:**

Psychological distress increases after a cancer diagnosis. However, patients who identify as Hispanic/Latino are less likely than their White counterparts to receive mental health care after being diagnosed, placing them at risk of poor psychological adjustment. The parent research objective aimed to characterize the treatment experiences of rectal cancer patients who identify as Hispanic/Latino. This qualitative secondary analysis specifically explored patients' emotional and psychological experiences.

**Methods:**

We partnered with local community health workers to recruit rectal cancer patients who identified as Hispanic/Latino and their caregivers from California's Bay Area and Central Valley. Semi‐structured interviews (*n* = 21) were conducted in either Spanish or English based on patient preference. Initial themes were identified using reflexive analysis. Experiences of distress were found in nearly all interviews, motivating this qualitative secondary analysis using directed content analysis.

**Results:**

We identified three main themes: (1) emotional reactions to the initial diagnosis, (2) perspectives and emotions experienced throughout treatment, and (3) coping strategies and advice for others. Participants reported shock and incongruence with their diagnosis. The treatment journey caused feelings of embarrassment and frustration from difficulties such as racial profiling and insurance. Patients coped through faith and support from trusted individuals.

**Conclusions:**

Rectal cancer patients who identify as Hispanic/Latino endure emotional distress at multiple stages of their cancer journey, including from sources that are unique to this population. Culturally and linguistically tailored interventions are needed to help mitigate this psychological burden and to provide active, coordinated support throughout the treatment phase in order to better prepare patients for survivorship.

## Abstract 2

Rectal cancer patients who identify as Hispanic/Latino endure significant distress at multiple stages of their cancer journey. Tailored interventions are needed to support the unique needs and coping strategies of this population.

## 1 Introduction

Colorectal cancer (CRC) is the third most common cancer among Hispanic/Latino (H/L) women and the second among H/L men. Although CRC has traditionally affected patients later in life, rates of early‐onset CRC have skyrocketed in the United States, with the largest increases occurring among H/L patients aged 20–29 [1, 2]. In addition to the human cost of these early‐onset diagnoses, a rising incidence among H/L patients presents challenges to cancer care since H/L patients are more likely to be diagnosed at an advanced stage and less likely to receive guideline‐concordant care [3, 4, 5].

Being diagnosed with cancer is associated with high levels of psychological distress, even after controlling for other sociodemographic risk factors [6]. Clinical depression is more common among patients with cancer (8%–24% vs. 7%–8% in the general population) and is more likely to have adverse effects on health, including reducing treatment adherence [7, 8]. H/L cancer patients, in particular, appear to be more likely to experience severe emotional distress [9] and less likely to seek and receive mental health services than cancer patients from other racial and ethnic groups [10]. Without appropriate psychosocial support, H/L cancer patients may be more susceptible to depression, more likely to experience disruptions in their quality of life and life roles, and less prepared for survivorship [11, 12, 13].

We performed a qualitative secondary analysis with two aims: (1) to explore how rectal cancer patients who identify as H/L and their caregivers experience the emotional distress of their diagnosis and cancer treatment and (2) to identify common sources of physical and emotional support. In so doing, we hoped to improve our understanding of cancer‐related psychological distress among H/L cancer patients and help inform future efforts within the field to develop culturally and linguistically appropriate tools to support these patients over the course of their disease.

## 2 Materials & Methods

### 2.1 Design

This analysis is a part of a larger study: “Advancing our Communal Understanding of Rectal cancer Disparities and identifying Opportunities for improvement” (ACUeRDO) [14]. During data analysis of the original study, it became apparent that participants had experienced significant psychological distress due to their diagnosis. We evaluated this area of interest by performing a qualitative secondary analysis. Our focus was: “What mental and psychological burdens do H/L rectal cancer patients experience and how are they coping with these burdens?”

#### 2.1.1 Design of Parent Study

The parent study was a qualitative, semi‐structured interview study conducted to elicit the experiences of H/L rectal cancer patients and their caregivers. The aim was to identify barriers and facilitators to care and to understand how these may differ based on rurality, social context, and community support. An interview guide was designed based on the Ecological Model of Health Behavior [15]. Topics covered included how participants experienced their initial diagnosis, how severe they initially perceived their disease to be, and what sources of support they relied on throughout their treatment. All participants provided consent to enroll in the study. The study was approved by the Stanford University Institutional Review Board (IRB #69543).

#### 2.1.2 Participants (Sampling & Recruitment)

Included patients had to (1) have received or be receiving treatment for rectal cancer, (2) be at least 18 years of age, and (3) identify as H/L. The parent study had a particular interest in patients living in either the San Francisco Bay Area or California's Central Valley to allow for comparisons based on rurality. Participants were directly recruited from Stanford Medicine medical oncology, radiation oncology, and colorectal surgery providers. Flyers were posted within these clinics for self‐referral. To explore a broader range of treatment experiences, we partnered with community health workers (promotoras) from Visión y Compromiso, a nonprofit organization committed to supporting community wellbeing, who utilized social media, broadcasting, and community events to recruit patients. Participants were encouraged but not required to bring a caregiver to the interview to gain a broader understanding of the impact of their cancer treatment on family and social functions. On occasion, caregivers were interviewed alone if the patient was unavailable or had passed away prior to the interview taking place.

#### 2.1.3 Data Collection

Two study team members (JH and EB) conducted 22 semi‐structured interviews between August 2023 and February 2024. Both were trained in conducting interviews, are proficient in Spanish, and have a medical background. Interviews were conducted in either English or Spanish and either in‐person or via Zoom, based on participant preference. Two follow‐up interviews were conducted after the participants expressed that they had more information to share. Interviews lasted 30–60 min. Participants were each given a $25 gift card to compensate them for their time. We received consent to audio record either through the Zoom platform or an encrypted device for in‐person interviews. Interviews were transcribed and de‐identified by a third‐party service. The same service translated Spanish‐language interviews.

#### 2.1.4 Data Analysis

The organization and coding of transcripts was accomplished using NVivo 14 (QSR International) for the parent and secondary analyses. The codebook began with deductive codes based on the interview guide and model framework. To create codes inductively, the two interviewers independently reviewed two transcripts. They discussed their initial codes, adjudicated where appropriate, and used this combined codebook to code two more transcripts. Inter‐rater reliability was reviewed using NVivo's Kappa score. For low‐performing codes, discrepancies were resolved through focused discussion, which included clarifying the codes' usage and meaning as well as conferring with other team members when necessary. As data analysis continued and new information was gathered, some codes were revised to capture the information more accurately. Edits included renaming and combining codes, as well as making minor changes to the definitions of the codes. When changes to the codebook were made, previously coded interviews were reviewed and recoded, if necessary. Inter‐rater reliability was assessed continuously during the process to ensure continued agreement [16, 17]. During analysis, one interview was excluded due to the participant not meeting the inclusion criteria (did not identify as H/L); therefore, 21 interviews were analyzed.

### 2.2 Qualitative Secondary Analysis

A secondary analysis of qualitative data is defined as using previously collected data to explore questions of interest that are different from the primary study's focus [18]. For this secondary analysis, we used a directed content analysis approach in which predefined codes are used to extend knowledge to a new context [19]. This method eliminated the need to recode the data as the inter‐rater reliability had already been assessed during the primary analysis. In the parent study, “Emotional State” was an inductive code that demonstrated strong agreement between the authors. As additional validation, transcript segments were iteratively re‐examined to ensure that the code was well‐defined and accurately represented the data and participant experiences. The experiences from this inductive exploration were then grouped to represent broader concepts, thereby providing a deeper understanding of psychosocial distress [20].

The primary author (ZC) was a member of the parent study team and had access to de‐identified and transcribed data, as well as the interviewers and primary investigator, for any clarifying questions. To ensure an understanding of the context and that no data was missing, all transcripts were reread in their entirety. If any inductive information was found and seemed relevant, the quote was copied into a table. Quotes were summarized, and concepts were compared to identify commonalities. Themes were inductively developed by grouping recurring sentiments into categories: (1) what emotions were experienced, (2) what caused these feelings, and (3) what are actionable ideas that can help others. To ensure credibility, findings were discussed among the study team, and NVivo's query tool was utilized to search for disconfirming evidence.

## 3 Results

### 3.1 Sample/Participant Demographics

Our final cohort included 16 patients and 7 caregivers. Of the 21 interviews, 14 were with patients alone, 3 were with a caregiver alone, and 4 were with both. Twelve interviews were conducted in Spanish, and the remaining nine were in English.

The majority of patients were 55 or older (68.8%). Among those who reported their education level, most had obtained a high school diploma (80.0%). All patients were diagnosed at least 6 months prior to enrollment, and 62.5% were receiving treatment. Nearly all patients were not working for varied reasons, including retirement (*n* = 5) or disability (*n* = 7). Most resided in the Bay Area (56.3%), and the remainder resided in the Central Valley. We did not collect demographic data from caregivers.

### 3.2 Themes

**FIGURE 1:**
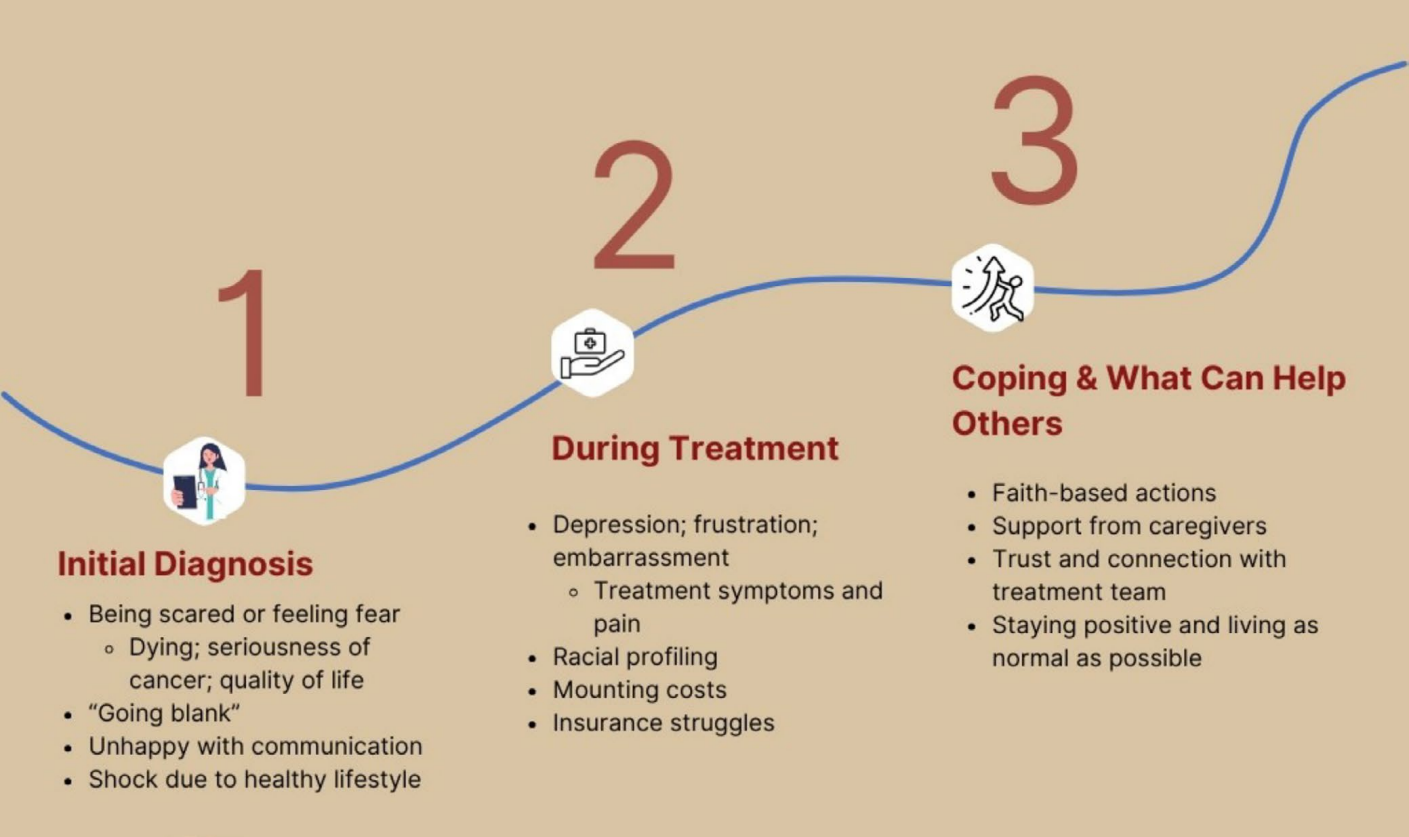
Overview of emotional reactions based on the phase of care.

Themes related to participants' cancer‐related distress included (1) emotional reactions to the initial diagnosis, (2) perspectives and emotions experienced throughout treatment, and (3) coping strategies and advice to others (Figure 1). Table 1 outlines participant demographics. Table 2 displays representative quotes addressing each of these themes.

**TABLE 1:** Participant demographics.

Patients	*N*=16
Gender	
Female	4 (25.0%)
Male	12 (75.0%)
Age, years	
25–54	5 (31.3%)
55–64	6 (37.5%)
65–74	4 (25.0%)
75+	1 (6.3%)
Race	
White	8 (50.0%)
Other	
Mexican	3 (18.8%)
Hispana	2 (12.5%)
Latino	2 (12.5%)
Caribbean	1 (6.3%)
Ethnicity	
Hispanic/Latino	16 (100%)
Marital status	
Single, never married	6 (37.5%)
Married or domestic partnership	8 (50.0%)
Divorced	2 (12.5%)
Highest degree or level of education	
Nursery school to 8th grade	3 (18.8%)
High School graduate, diploma, or equivalent	1 (6.3%)
Some college credit, no degree	2 (12.5%)
Associate Degree	1 (6.3%)
Bachelor Degree	7 (43.8%)
Graduate Degree	1 (6.3%)
Prefer not to say	1 (6.3%)
Employment status	
Employed for wages	2 (12.5%)
Out of work and looking for a job	1 (6.3%)
Out of work and not looking for a job	1 (6.3%)
Unable to work	7 (43.8%)
Retired	5 (31.3%)
Location	
Bay Area	
Santa Clara	4 (25.0%)
San Francisco	1 (6.3%)
San Mateo	2 (12.5%)
Santa Cruz	2 (12.5%)
Central Valley	
Stanislaus	1 (6.3%)
Merced	4 (25.0%)
San Joaquin	2 (12.5%)
Received their rectal cancer diagnosis	
Last year	5 (31.3%)
Last 2 years	6 (37.5%)
Last 5 years or longer	5 (31.3%)
Currently receiving treatment	
Yes	10 (62.5%)
No	6 (37.5%)
Caregivers^a^	7

^a^ Demographic information was not collected for caregivers.

**TABLE 2:** Representative quotes related to each theme.

Theme	Quote
#1: Initial Diagnosis	Well, at first, it was kind of hard to take it in when the doctor told me I had the cancer. Well, he told me it was a tumor, and I just kind of went blank. (ID 7, Male, Patient, English)
	Well, basically I thought I was going to die. But to be honest, I thought my quality of life was the one that was going to be affected because when I was diagnosed, I was only… I remember 24 days into being married. I married on November 1st, and on the 24th, I was diagnosed with cancer. So for me, when he told me they're going to have a bag, I immediately thought my life, my work, my life with my husband, and so on, you know, like so for me was the quality of life was going to be really, really affected. And of course, you… you're scared about you know dying, but it was more about the quality of life. (ID 1, Female, Patient, English)
	He said you have cancer. I was very worried, I said cancer, how will that be? How am I going to heal? So many concerns. (ID 21, Male, Patient, Spanish)
	I felt healthy, I never hurt at all, I was a person who always walked around a lot. …. so how was I going to imagine that this was happening to me inside …. in my family, there is no one who has it [cancer]. I am the first. (ID 23, Male, Patient, Spanish)
	Yes, I had knowledge [of cancer], but I never imagined that it would have happened to me, because I have been a healthy man, I was a soccer player. (ID 11, Male, Patient, Spanish)
#2: During Treatment	It's like they're staring at me and I'm feeling like they're seeing the ball I have in my stomach…. Yes, it feels uncomfortable. When they look at you funny, because they think you have something in your belly, or when I go to the stores, they think like I put something in my belly, that I stole something. (ID 12, Male, Patient, Spanish)
	When I first arrived, when I said, oh my God, I don't have insurance, they want to send me to (name of the hospital), and I don't have any. (ID 4, Female, Patient, Spanish)
	It's been frustrating for me not to be out of my illness. Right? It's frustrating because when I go to the bathroom, I have trouble. It's frustrating, to sit and try to defecate and not be able to and feel that pain, that's it, it's ugly, horrible. Right? That's frustrating, and another thing, frustrating, is that when, perhaps, you find a doctor who doesn't treat you well, a nurse, who doesn't treat you well. (ID 11, Male, Patient, Spanish)
	Thank God, everything has gone very well for me, and thanks to the fact that I had a good one, I feel that I had a good treatment and good advice from the doctors, from everyone. (ID 22, Male, Patient, Spanish)
	I think the support of the oncologist and the explanation and the treatment, because I started with chemotherapy, and the environment in which I was, I think everything was positive. (ID 16, Male, Patient, Spanish)
#3: Coping & Actionable Ideas	I feel like a peace within myself, like sharing with all the ladies who go to that [prayer] group. Something takes away the stress that I may feel. I'm sad, I go there and talk, and I feel a little better. (ID 24, Female, Caregiver, Spanish)
	And with the hope of God, the promise He has given us. So that's helped me a lot. I'm very calm about the disease …. I'd like to say to a person starting treatment. Ah. Don't despair, trust God, trust doctors. There are good treatments to be able to survive. Don't lose hope. (ID 14, Male, Patient, Spanish)
	You know it makes you think if your body's telling you something is wrong, you should go to the doctor right away….She told me, actually, too… She had been very tired. That was one of the big things that she noticed….She always had problems. Going to the restroom, pain in her stomach. But you know, we, I think, we Latins are very stubborn …we're dying. We don't go see the doctor. (ID 18, Female, Caregiver, English)
	I know there's a lot of patients and it's a… it's a job, you know, it gets to a point that it's a job, but like take more … a little bit more time and ‘This is going to happen, and you know you're going to experience this’ [ID 1 speaking slowly and calmly] versus ‘OK, this is going to happen boom, boom, boom’ [ID 1 speaks faster and hurried]. (ID 1, Female, Patient, English)
	(When asked what type of support family provides): Emotional [support] and then just being here at the house, cooking for her, helping me with feeding her, cleaning her up. … Just to make sure she was OK…that she had what she needed at home… (ID 2's Son, Male, Caregiver, English)

#### 3.2.1 Emotional Reactions to the Initial Diagnosis

Respondents described experiencing significant psychosocial distress during the initial diagnosis. Participants' most common reactions were fear and uncertainty. Thirteen participants wondered if they were going to die, were afraid because they knew that cancer is a serious diagnosis, and worried about their quality of life and how life would be changing for them. The uncertainty was summarized by one patient:

Yes [it was scary to hear you have to have chemotherapy] because you don't know what the treatment will be like…. You start thinking, what is this all going to be like? (ID 15, Male, Patient, Spanish)

Two participants also felt that their initial diagnosis was not delivered with care, which added to their distress; they remembered the experience as being harsh or hurried. Five patients also discussed dissociation or “going blank” when being diagnosed. The most reported example of “going blank” was not hearing the doctor or being unable to remember certain words from the conversation: “You hear things, but you don't listen. It's like a blur.” (ID 1, Female, Patient, English) Four patients also mentioned being shocked at their diagnosis because it seemed discordant with their healthy lifestyles or being confident that their symptoms were related to a less serious diagnosis, like hemorrhoids: “A surprise… I took care of myself a lot, and I didn't expect it.” (ID 17, Male, Patient, Spanish) Another patient explained further: “I exercised, I ate well, I didn't eat much processed food… So, when they gave me the news, I felt like maybe they were confused.” (ID 14, Male, Patient, Spanish)

Caregivers also experience unique burdens at the time of the initial diagnosis that may go unacknowledged. Two caregivers recalled how difficult it was to be the ones to convey the diagnosis to the patient after he or she awoke from his or her colonoscopy; one summarized the ordeal:

I would describe it as a traumatic experience …and the added layer of me actually having to be the one to tell my husband what had been discovered…. it was quite devastating, traumatic, and lonely. (ID 6, Female, Caregiver, English)

#### 3.2.2 Perspectives and Emotions Experienced Throughout Treatment

Patients reported feeling depressed, frustrated, and embarrassed during their treatment due to symptoms and various logistical difficulties they experienced. Symptoms included bleeding and severe abdominal pain. Six participants reported they were not able to work due to symptoms, which contributed to the emotional burden: “I do get depressed…. Because I can't work, I can't walk freely, I'm in pain.” (ID 15, Male, Patient, Spanish)

Additional sources of psychological distress during treatment included racial profiling, a lack of consistent social support, and dealing with their health insurance. Two participants described experiencing racial profiling. One patient felt a store clerk suspected he was stealing due to the bulge under his shirt from his ostomy bag. A caregiver and patient pair also recalled a time when they were refused care as the clinic staff did not believe they could afford the payment for the visit: “It was a girl who turned us away. I said, I don't know why, but I'm sure they think we don't have the money to pay.” (ID 26, Caregiver, Female, Spanish) Patients also commented on how the lack of social support added to the difficulty of going through treatment. One participant stated how difficult it was to adhere to her appointments during the COVID‐19 pandemic restrictions because she felt lonely without her family. Another participant expressed that his mounting treatment costs and conflicting information from his provider created an “emotional roller coaster” to the point where he did not want to continue treatment. Conversely, patients who felt more supported by providers recalled feeling “happy” and having a more positive experience during treatment. As recalled by one patient, “I was kinda scared but after talking to the doctor…He explained everything to me…I was a little confident with him – real relaxed with him.” (ID 7, Male, Patient, English)

Three participants identified health insurance as a source of stress during their treatment journey. One patient feared she would not receive insurance because she was undocumented at the time of diagnosis. A caregiver stated that she felt a “complete and utter loss of power” when dealing with her insurance company. She recalled a time when her husband had begun preparing for a procedure only to be told the day before that he could no longer have it because the insurance company had deemed it unnecessary: “It's really that stuff that adds suffering to the suffering…. You're already lying on the ground, and somebody kicks you in addition.” (ID 6, Female, Caregiver, English)

#### 3.2.3 Coping Strategies for Management of Negative Emotional States and Advice to Others

Faith and religion were nearly universally mentioned as important sources of support. Eighteen participants reported that faith‐based activities, such as trusting in God, utilizing prayer or scripture, and receiving support from other believers, brought them tranquility, peace, and encouragement. One caregiver declared, “If I didn't have faith or didn't have anything to lean on, what would I be?” (ID 24, Female, Caregiver, Spanish)

Support from caregivers was also mentioned as a primary source of support, with the caveat that caregiver burnout is a real and significant risk. Seventeen participants told stories of family and friends offering emotional, informational, and tangible support through encouragement, sharing their own cancer experiences, and transporting patients to and from their appointments. However, one patient mentioned that rectal cancer is a unique experience that is not as openly discussed: “I really didn't have any other people that had rectal cancer that could explain it to me or tell me this is what you're gonna go through.” (ID 13, Male, Patient, English) Others felt taxed by watching a family member go through the treatment process and identified that as a source of emotional burden. One caregiver specifically discussed the risk of burnout:

As a caregiver, ….You're the one who needs to be fine, so take care of yourself …. Both of us need to maintain this motivation to keep going on so many levels. (ID 6, Female, Caregiver, English)

For ten participants, trust and personal connections with the treatment team helped them stay engaged and contributed to feeling supported throughout their treatment journey. As summarized by one patient: “Cancer tires you, but the company of the doctors, …the interpreter they give you…you feel like family, and it helps you a lot.” (ID 17, Male, Patient, Spanish) When asked what could be done to improve their experience, participants mentioned ways to improve communication with the treatment team, including being more sensitive when conveying the initial diagnosis, explaining complex treatment concepts in layman's terms, and having an interpreter at each clinic visit.

Overall, participants described coping strategies as protective but often incomplete. One participant spoke highly of the support he received from his family and faith but expressed a sense of weariness after describing his symptoms and his treatment course, which included multiple invasive procedures: “We'll see how long I can take this.” (ID 11, Male, Patient, Spanish) Patients recommended combating the psychological distress of their diagnosis by staying positive, moving forward, and continuing to live their lives. One patient remarked that getting back to “regular life” in between his chemotherapy appointments was beneficial: “It would keep my spirits up…it was giving me something to look forward to get through the 3 days [of chemo] to be like let's go eat pizza after this.” (ID 3, Male, Patient, English) Another patient shared that staying busy in talks and activities with trusted people helped him stay positive and combat his depression.

## 4 Discussion

In this secondary analysis of a qualitative cohort study, H/L patients with rectal cancer reported psychosocial distress from a variety of sources throughout their treatment journey. Participants reported feeling shocked and “going blank” during their initial diagnosis. Their treatment journeys were sometimes marred by racial profiling, worrying about whether their documentation status would affect treatment, and stress from dealing with insurance companies, which left them feeling depressed and embarrassed. Most patients coped through faith and support from their family, friends, and treatment team.

Our results are congruent with previous literature on the topic. A similar study of CRC patients/caregivers in Australia acknowledged the treatment team as a significant source of support and categorized the forms caregiver and treatment team support can take [21]. Another qualitative study of Hispanic and non‐Hispanic Black cancer patients described their initial diagnosis and treatment as significantly disruptive to their lives. Many participants reported mental health problems (e.g., anxiety and post‐traumatic stress disorder) and treatment barriers, including a lack of insurance coverage and poor access to mental health support [22]. Although similar, neither previous study specifically explored the experiences of H/L CRC patients. Our study, therefore, contributes to literature by identifying unique challenges (e.g., racial profiling) and sources of support (e.g., faith/religion) that are of importance to this community.

Our findings have several important implications for better addressing the emotional needs of H/L patients with rectal cancer. First, our results align with prior literature regarding the need to tailor psychological interventions to the community at risk [23, 24, 25]. Although psychosocial interventions appear to improve outcomes for cancer patients in general [26, 27, 28], there is little extant data on interventions to support H/L cancer patients; the few studies that exist focus on improving screening rates or transitioning from treatment to survivorship [29, 30]. Our study, therefore, provides new information to help direct future interventions. Our results indicate that patients' emotional distress begins at diagnosis and continues through treatment and into survivorship, suggesting that interventions should be targeted earlier in patients' treatment journeys. This finding is also supported by previous literature on early‐stage psychosocial interventions to reduce distress [31, 32, 33]. In addition, at least some of the barriers faced by H/L rectal cancer patients appear to be unique, even compared with H/L patients with other types of cancer, given the sensitive nature of the symptoms and the potential need for an ostomy. Continued research into the challenges that specific groups of cancer patients face undoubtedly will help inform the development of more efficient, more culturally concordant, and, hopefully, more effective interventions.

Second, our results suggest that there may be significant gaps in knowledge regarding CRC and its presenting symptoms, at least among the H/L rectal cancer patients in our study. Multiple patients reported disbelief when presented with their diagnosis since they thought of themselves as having lived a healthy lifestyle. One patient even mentioned that he never had a colonoscopy because he had never had any symptoms. Given the rapid increase in early‐onset CRC among H/L patients, primary care providers, public health officials, and community leaders need to emphasize the importance of staying up to date on CRC screening and reporting symptoms when they occur.

Third, we also identified significant opportunities for improvement in how cancer diagnoses are being delivered to patients and their caregivers. The American Society of Clinical Oncology has developed best practice guidelines for patient–clinician communication, including ensuring that the timing and privacy of the setting are appropriate for the discussion and helping patients find additional sources of support when delivering bad news [34]. Interestingly, there is no mention of ensuring clinicians convey the diagnosis. Placing that burden on the caregiver or loved one—as was the case in several of our interviews—is an enormous emotional burden and can contribute to significant psychological distress. Based on our results, it may also be advantageous for providers to have faith‐based resources available, as they appear to be a major source of support within the H/L community.

Fourth, caregivers experience similar psychological and emotional burdens to patients, putting them at risk of developing mental health conditions. In our study, caregivers reported experiencing sadness and grief at the initial diagnosis, stress from dealing with insurance companies and arranging transportation during treatment, and the added weight of being the primary source of motivational support. There are few studies addressing psychological interventions for caregivers, although one reported lower levels of stress and fatigue after implementing a mindfulness intervention with H/L CRC patients and their caregivers [35]. Given that an estimated 25% of Americans report caring for a family member or friend with serious illnesses [36], recognizing and addressing psychosocial burden among caregivers may have more significant implications for improving mental health in the U.S. than interventions directed at cancer patients alone.

Finally, high levels of psychological distress and inadequate psychosocial support can contribute to negative oncologic outcomes. Prior studies have identified depression and anxiety as significant predictors of both late initiation of treatment and treatment nonadherence, suggesting that improving cancer patients' mental health may also affect their prognosis [37, 38, 39]. Participants in our study who described poor social support, concerns regarding treatment costs, and inconsistent or confusing information from providers also reported being less willing to attend their appointments or to continue treatment. Given our findings that H/L cancer patients rely heavily on family and friends for physical and emotional support and that caregiving increases the risk for burnout, the true effects of psychological distress on treatment and clinical outcomes may be magnified if caregivers can no longer provide the necessary support [38, 40, 41].

The study has several limitations. First, our sample is small, commensurate with qualitative methodology, and many patients received care at the same academic cancer center. Our results, therefore, may not extend to other populations or treatment settings. However, the majority of participants received care at smaller centers before being referred to Stanford and were interviewed about their experiences during their entire cancer journey. Moreover, rectal cancer is often regionalized to high‐volume academic centers, given the complexity of treatment options. Second, most participants in our sample were older, and our findings may not be generalizable to early‐onset CRC patients. Given the sharp increase in early‐onset CRC within the H/L community, we maintain there is an urgent need for similar studies to explore the experiences of early‐onset CRC patients and their caregivers so that interventions can be designed to better support their potentially unique psychosocial needs. Finally, as with any qualitative study, there is a risk of investigator bias in interpretation. To mitigate this risk, the data was examined in multiple iterations, findings were verified across the study team, and disconfirming perspectives were sought for every major finding.

In conclusion, H/L rectal cancer patients reported emotional burdens throughout their treatment, including dissociation from the diagnosis, depression, insurance concerns, and being subjected to racial profiling. Patients coped via faith and support from family, friends, and providers. We believe that our results can inform culturally and linguistically tailored interventions to help provide psychosocial support to both H/L rectal cancer patients and their caregivers throughout their cancer journeys.

## Author Contributions

**Zaria N. Cosby:** formal analysis (lead), investigation (equal), methodology (lead), writing – original draft (lead), writing – review and editing (lead). **Julian P. Howland:** conceptualization (equal), data curation (equal), formal analysis (equal), project administration (equal). **Eleanor Brown:** conceptualization (equal), data curation (equal), formal analysis (equal), investigation (equal), project administration (equal), writing – review and editing (equal). **Lucas K. Carpenter:** conceptualization (equal), data curation (equal), formal analysis (equal), project administration (equal), writing – review and editing (equal). **Kristen M. Davis‐Lopez:** conceptualization (equal), formal analysis (equal), methodology (equal), writing – review and editing (equal). **Min Young Kim:** data curation (equal), formal analysis (equal), investigation (equal), methodology (equal), writing – review and editing (equal). **Patricia Castañeda:** conceptualization (equal), investigation (equal). **Maria Gonzalez:** conceptualization (equal), investigation (equal). **Miriam T. Hernandez:** conceptualization (equal), investigation (equal). **Ysabel Duron:** conceptualization (equal), investigation (equal). **Gladys M. Rodriguez:** conceptualization (equal), investigation (equal). **Sandra S. Zaky:** conceptualization (equal), investigation (equal), methodology (equal), writing – review and editing (equal). **Arden M. Morris:** conceptualization (equal), investigation (equal), methodology (equal), supervision (equal), writing – original draft (equal), writing – review and editing (equal). **Aaron J. Dawes:** conceptualization (equal), funding acquisition (equal), investigation (equal), methodology (equal), project administration (equal), resources (equal), supervision (equal), writing – original draft (equal), writing – review and editing (equal).

## Conflicts of Interest

The authors declare no conflicts of interest.

## Data Availability

The data that support the findings of this study are available on request from the corresponding author. The data are not publicly available because of privacy or ethical restrictions.
